# Meta-Analysis of *Aedes aegypti* Expression Datasets: Comparing Virus Infection and Blood-Fed Transcriptomes to Identify Markers of Virus Presence

**DOI:** 10.3389/fbioe.2017.00084

**Published:** 2018-01-11

**Authors:** Kiyoshi Ferreira Fukutani, José Irahe Kasprzykowski, Alexandre Rossi Paschoal, Matheus de Souza Gomes, Aldina Barral, Camila I. de Oliveira, Pablo Ivan Pereira Ramos, Artur Trancoso Lopo de Queiroz

**Affiliations:** ^1^Instituto Gonçalo Moniz, Fundação Oswaldo Cruz (FIOCRUZ), Salvador, Brazil; ^2^Post-Graduation Program in Biotechnology in Health and Investigative Medicine, Fundação Oswaldo Cruz (FIOCRUZ), Salvador, Brazil; ^3^Federal University of Technology—Paraná, UTFPR, Campus Cornélio Procópio, Cornélio Procópio, Brazil; ^4^Federal University of Uberlândia, Patos de Minas, Brazil; ^5^Post-Graduation Program in Health Sciences, School of Medicine, Federal University of Bahia, Salvador, Brazil; ^6^Post-Graduation Program in Applied Computation, Universida de Estadual de Feira de Santana, Feira de Santana, Brazil

**Keywords:** *Aedes aegypti*, alimentation, blood-feeding, meta-analysis, transcriptomics, vector-borne diseases, virus infection

## Abstract

The mosquito *Aedes aegypti* (L.) is vector of several arboviruses including dengue, yellow fever, chikungunya, and more recently zika. Previous transcriptomic studies have been performed to elucidate altered pathways in response to viral infection. However, the intrinsic coupling between alimentation and infection were unappreciated in these studies. Feeding is required for the initial mosquito contact with the virus and these events are highly dependent. Addressing this relationship, we reinterrogated datasets of virus-infected mosquitoes with two different diet schemes (fed and unfed mosquitoes), evaluating the metabolic cross-talk during both processes. We constructed coexpression networks with the differentially expressed genes of these comparison: virus-infected versus blood-fed mosquitoes and virus-infected versus unfed mosquitoes. Our analysis identified one module with 110 genes that correlated with infection status (representing ~0.7% of the *A. aegypti* genome). Furthermore, we performed a machine-learning approach and summarized the infection status using only four genes (AAEL012128, AAEL014210, AAEL002477, and AAEL005350). While three of the four genes were annotated as hypothetical proteins, AAEL012128 gene is a membrane amino acid transporter correlated with viral envelope binding. This gene alone is able to discriminate all infected samples and thus should have a key role to discriminate viral infection in the *A. aegypti* mosquito. Moreover, validation using external datasets found this gene as differentially expressed in four transcriptomic experiments. Therefore, these genes may serve as a proxy of viral infection in the mosquito and the others 106 identified genes provides a framework to future studies.

## Introduction

The mosquito *Aedes aegypti* (L.) is the main vector of dengue virus (DENV), West Nile virus (WNV), and Yellow fever virus (YFV), present worldwide (Mackenzie et al., 2004; Lorenzo et al., 2014); more than 2.5 billion people in over 100 countries are at risk of contracting dengue alone (World Health Organization, 2015), while yellow fever remains endemic in tropical regions of Africa and South America (Bae et al., 2005). West Nile fever, despite causing occasional small outbreaks, shows an extremely high mortality rate (Pradier et al., 2012). Beyond established pathogens, other viruses are also on the rise as public health problems: chikungunya virus, formerly restricted to parts of Africa, is now globally spread (Cauchemez et al., 2014). Zika virus has recently become a global concern, after initial outbreaks in the Pacific region in 2007, followed by a larger spread in the Americas (Roth et al., 2014; Zanluca et al., 2015), including Brazil (Morens and Fauci, 2014; Petersen et al., 2016; Slavov et al., 2016). Despite extensive vector control measures to curb transmission, including source reduction, pesticides, public education and biological control, these efforts were largely unsuccessful (Medlock et al., 2012), highlighting the need for enhanced control methods and better knowledge about mosquito biology (Seixas et al., 2013; Porse et al., 2015).

In 2007, the *A. aegypti* complete genome was released (Nene et al., 2007) and vector-specific databases were developed, such as Vector Base (Giraldo-Calderon et al., 2015). This allowed expression assays addressing viral infection (Colpitts et al., 2011), enabling new insights about the *A. aegypti* gene regulation and transcriptional processes (Dissanayake et al., 2010; Colpitts et al., 2011). Further studies focused on the gene expression profile related to mosquitoes blood feeding (females) compared to nonblood-fed (N-BF, males), suggesting that sex- and stage-specific genes play an important role on the feeding response (Dissanayake et al., 2010). During feeding, female mosquitoes acquire blood that is necessary for egg development and may subsequently become infected with pathogens. Both infection and blood feeding processes induce changes in gene expression levels. The intrinsic physiological crosstalk between these process are linked and a joint analysis is required to assess patterns of infection possibly unappreciated in previous studies, due to the difficulty of separating the gene expression patterns that arise from feeding on blood from that resultant of the infection process due to host–pathogen interactions.

To address this issue, we performed an integrated gene expression analysis of currently available data sets. One dataset is derived from mosquitoes infected with DENV, WNV, YFV, or uninfected and another from blood-fed (BF) or sugar-fed *A. aegypti*. We identified 110 genes specifically associated with an infection expression profile. Following data mining, we propose three main candidate genes (*AAEL014210, AAEL002477*, and *AAEL005350*) that relate to the infection caused by each virus and one gene, *AAEL0012128*, able to summarize the infection profile.

## Materials and Methods

### Description of *A. aegypti* Discovery Dataset

We jointly analyzed two previously published microarray datasets, available from the GEO under accession n. GSE28208 and GSE22339. The Colpitts et al. (2011) dataset (GSE28208) reports *A. aegypti* mosquitoes (Rockefeller strain) artificially infected through intrathoracic inoculation with DENV (type 2), YFV, or WNV and uninfected controls sugar-fed with raisins. The Dissanayake et al. (2010) dataset (GSE22339) reports *A. aegypti* mosquitoes [Liverpool (LVP) strain] that were strictly sugar-fed with raisins or that, besides having access to sugar feeding with raisins, were also BF on anesthetized mice. That study investigated the differential gene expression during the feeding process. From the total of 61 gene expression samples reported by both studies, we used a subset of 58 samples in our combined analysis, as discovery dataset. Table 1 summarizes the main characteristics of these samples.

**Table 1:** Description of *A. aegypti* studies used in the integrative analysis.

Dataset	Characteristics (infection status, sex)	Group classification	Number of samples
GSE28208 (Colpitts et al.)	DENV infected, nr	Infected	9
	YFV infected, nr	Infected	10
	WNV infected, nr	Infected	9
	Uninfected, nr	uninfected	9

GSE22339 (Dissanayake et al.)	Uninfected, ♀	BF	18
	Uninfected, ♀	N-BF	3

*♀, female; nr, sex not reported; DENV, dengue virus; WNV, West Nile virus; YFV, yellow fever virus; BF, blood-fed; N-BF, nonblood-fed*.

To facilitate reading, in what follows we refer to the group of uninfected mosquitoes that did not receive blood as N-BF and to the group of uninfected mosquitoes that had access to a blood meal as BF. These are samples from GSE22339 dataset. The groups of mosquitoes infected by any of the three included viruses (DENV, WNV, or YFV) are referred to as “infected,” independent of their feeding regimen and the uninfected mosquitoes were referred as uninfected. These are samples from GSE28208 dataset. In summary, our discovery dataset was composed by 28 virus-infected (DENV, YFV, and WNV) samples, 27 fed (18 with blood and 9 uninfected) and 3 N-BF uninfected samples.

### Data Collection, Preprocessing and Correction

Raw expression data from the 58 samples in both studies were downloaded from the GEO database.^1^ Quantile normalization was applied using the *preprocessCore*R package (R 3.2.2, R Foundation, Vienna, Austria). Only probes mapping to genes common to both datasets were kept. Since we performed a joint analysis of two different datasets of interest, the expression data was submitted to a correction procedure using an empirical Bayes framework implemented in the COMBAT tool (Johnson et al., 2007). COMBAT corrects for experimental variation, commonly known as batch effects. Combining microarray data sets makes it possible to increase statistical power when detecting biological phenomena from diverse experiments. The present samples were classified into two batches according to the origin of each dataset (either GSE22339 or GSE28208), resulting in a merged dataset with corrected expression values. After merging and batch effect correction, the expression table was log2-transformed and differentially expressed genes (DEGs) were identified using an absolute log2-fold-change threshold of ≥1.0, and *t*-tests comparisons were performed with the Benjamini–Hochberg false discovery rate adjustment for multiple testing set at 5%.

### Coexpression Network Analysis

Weighted gene correlation network analysis (WGCNA) methodology was applied in the DEGs to construct a gene coexpression network with weighted interactions (Langfelder and Horvath, 2008). The biweight mid correlation algorithm implemented in the *bicor* function in WGCNA was used as correlation metric to compare gene expression values, being similar to Pearson’s statistic but is more robust to outliers (Langfelder and Horvath, 2012). In the WGCNA framework, the correlation matrix is transformed into a weighted adjacency matrix by applying a power transformation, f(*x*) = *x*^β^, where β is chosen such that the topology of the obtained adjacency matrix is approximately scale-free. Herein, the appropriate β parameter was set to 7. The scale-free model fitting index using this parameter was *R*^2^ = 0.85. Next, a topological overlap matrix (TOM) was derived from the adjacency matrix, taking into account gene expression connectivity. 1-TOM was used as a dissimilarity metric for hierarchical clustering and the detection of coexpression modules. The dynamic tree cut algorithm within WGCNA, set at default parameters, was used for module assignment. Module eigengenes (MEs), the first principal component of all gene expression values in a module, which summarizes expression values in a given module, were tested with respect to associations with the traits of interest (infected by each virus, BF and N-BF), and those found to be significantly correlated (absolute Pearson’s *r* ≥ 0.6; *p*-value < 0.05) were further studied by means of functional analysis using Gene Ontology (GO) terms.

### Functional Analysis Using GO Terms

Functional analysis of the significant genes were assessed by mapping GO terms extracted from Vector Base^2^ (Giraldo-Calderon et al., 2015). GO provides a controlled vocabulary of molecular functions, biological processes and cellular localization. Revigo was used to summarize the GO terms, employing *Drosophila melanogaster* as a reference (Supek et al., 2011).

### Data Mining

Decision trees were employed to identify a minimal set of gene expression measurements allowing separation between the infected from uninfected groups. This method analyzes all the phenotypic attributes (gene expression measurements) and selects the most relevant attributes that allow group classification (Sathler-Avelar et al., 2016). As input for tree construction, we used the 110 genes (and their expression values) that we identified as most related to infection independent of feeding background (available in Table S1 in Supplementary Material). The J48 algorithm implemented in the WEKA program (Waikato Environment for Knowledge Analysis, version 3.6.11, University of Waikato, New Zealand) was used to build a decision tree using default parameters (Espíndola et al., 2015). To estimate the classification accuracy of the decision tree models, we employed a 10-fold cross validation methodology. This methodology splits the dataset in a training set and testing set. The partition procedure is applied to avoid bias in sampling of training/test sets. Thus, the training set was used to tune the parameters, learning and building a model. The validation set was used to test the performance of the classifier in an unbiased way. The sensibility and specificity were measured from the confusion matrix and the receiver–operating characteristic curve (ROC).

### Hierarchical Clustering and Principal Component Analysis (PCA)

Hierarchical clustering was performed with genes with significant expression differences using Euclidean distance as a measure of dissimilarity and average linkage for between-cluster separation (*hclust* function in the *stats* package in R 3.2.2). Heatmap was generated in R *via* the *heatmap.2* function in the *gplots* package, using the “scale = ‘row’” switch to Z-score standardize the rows. The Z-score standardization measures the expression level of a gene in terms of number of SDs from the mean expression of the gene in all compared samples. PCA was performed in R 3.2.2 (function *prcomp*) in order to compare and visualize the grouping between infected, BF and N-BF samples using the gene expression data as input. The graphing package *ggplot2* (Wickham, 2016) was used to plot these results.

### Description of Validation Dataset

Expression data from the studies of Behura et al. (2011), Sim and Dimopoulos (2010), Bonizzoni et al. (2011), and Bonizzoni et al. (2012) available in GEO (accession nos. GSE16563, GSE33274, GSE24872, and GSE32074, respectively) were used to evaluate whether the same expression pattern observed in gene *AAEL012128* in our results could be verified in independent datasets, since they were not used in the Discovery step. GEO2R was used to access this gene expression in each validation dataset. In summary, the total samples used in validation were 16 samples infected with DENV and 12 fed samples.

## Results

### Blood-Feeding Triggers Differential Gene Expression Compared to Strictly Sugar-fed *A. aegypti*

**Figure 1:**
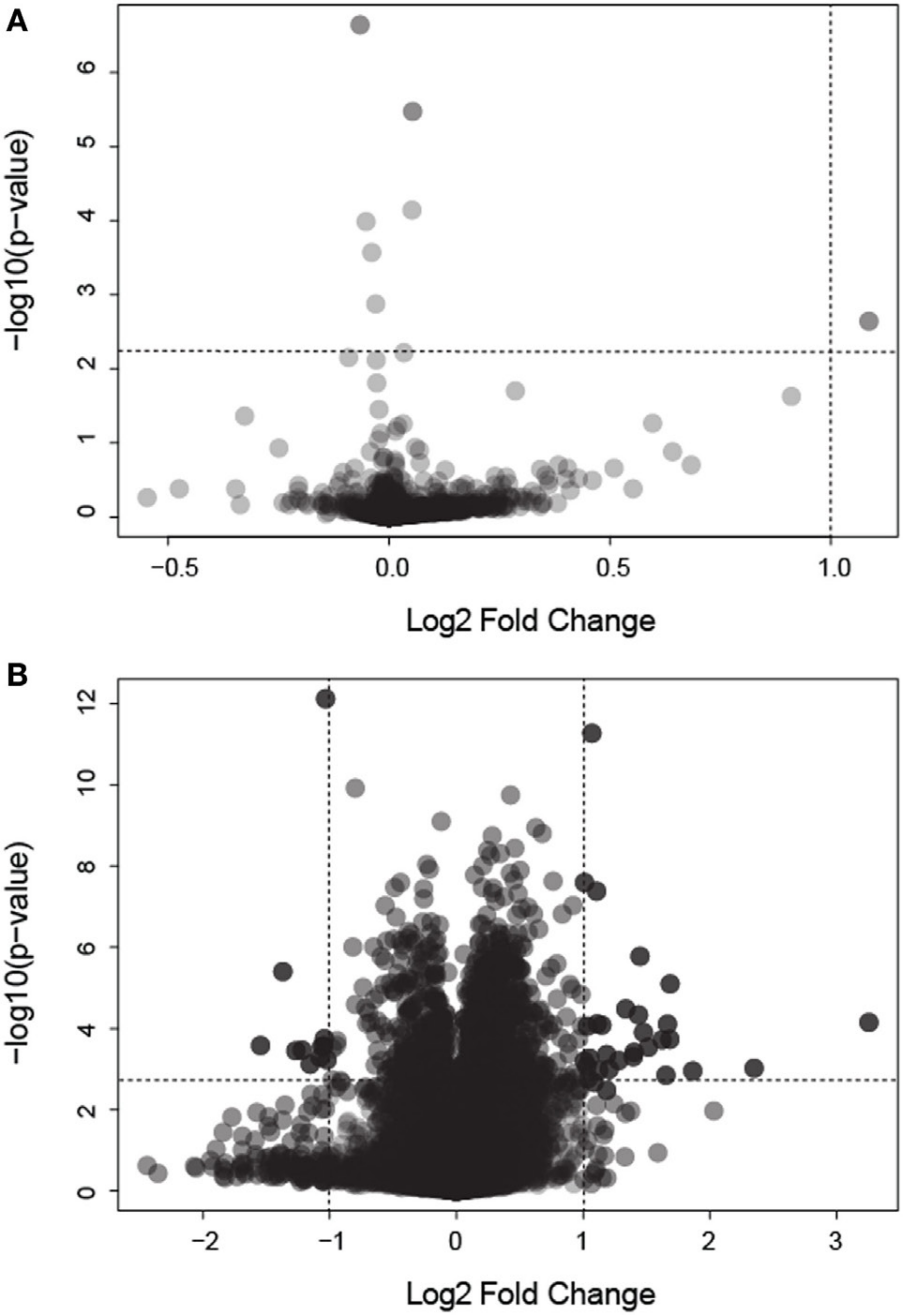
Volcano plot depicting differentially expressed genes (DEGs) in mosquitoes submitted to different feeding regimens. **(A)** Comparison of blood-fed (BF) vs. uninfected mosquitoes. **(B)** Comparison of BF vs. non-BF mosquitoes. All data were log2 transformed. Dotted lines represent the significant *p*-value and fold change cutoffs used to identify DEGs (see Materials and Methods).

We analyzed gene expression data from 58 samples stemming from two different studies, the Colpitts et al. (2011) and the Dissanayake et al. (2010) datasets. We merged both datasets, correcting for batch effects, and reanalyzed them with a focus toward the investigation of DEGs in: (i) BF vs. uninfected mosquitoes and (ii) BF vs. N-BF mosquitoes (Figure 1). BF and uninfected mosquitoes showed a similar gene expression pattern, without any differentially modulated genes (Figure 1A). However, comparison of BF vs. N-BF mosquitoes enabled the identification of 42 DEGs (32 upregulated and 10 downregulated genes) (Figure 1B).

### The Global Perturbation of Virus Infection Is Influenced by Feeding Status

**Figure 2:**
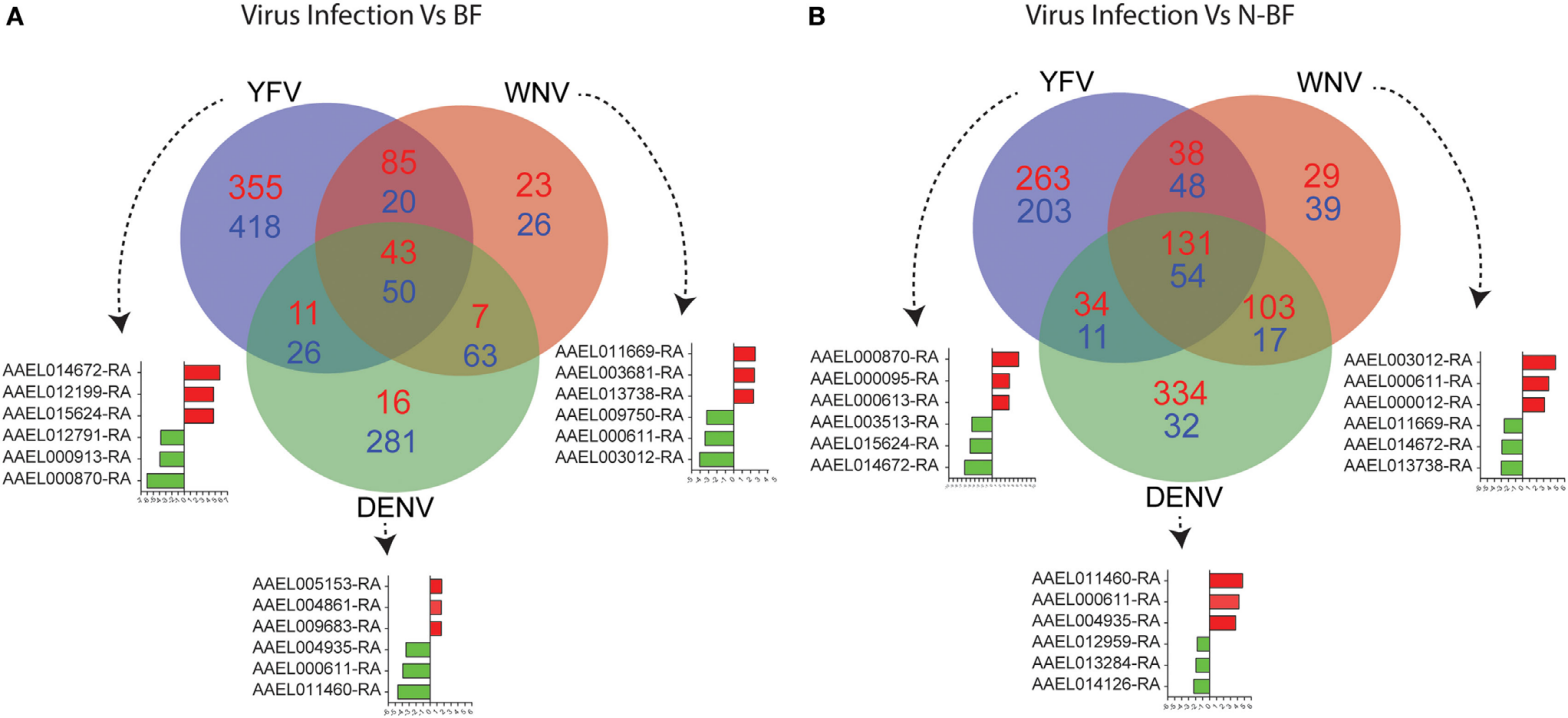
Critical signature differences in the distinct feeding schedules. **(A)** Differentially expressed genes (DEGs) from the comparison of genes from virus infected mosquitoes [yellow fever virus (YFV), dengue virus (DENV), and West Nile virus (WNV) vs. blood-fed (BF)]. **(B)** DEGs from the comparison of genes from virus infected mosquitoes (YFV, DENV, and WNV vs. non-BF). The blue numbers represent downregulated genes and the red numbers represent upregulated genes by absolute log_2_-transformed fold-change ≥1 criterium. Red and green bars correspond to up- and downregulated genes, respectively.

The DEGs identified in BF vs. N-BF mosquitoes is indicative that the feeding process modulates gene expression. Next, we evaluated DEGs during infection caused by DENV, YFV, and WNV compared to BF and N-BF mosquitoes (Figure 2). In the first part, we evaluated whether infection could drive differences in gene expression. For this, we compared infected mosquitoes separately vs. BF and 725 genes were found to be downregulated; YFV (*n* = 418 genes), WNV (*n* = 26), and DENV (*n* = 281). The intersection between all infection conditions showed a greater proportion of downregulated genes (*n* = 50), compared to upregulated (*n* = 43) (Figure 1A). In the comparison of infected (DENV, YFV, and WNV) vs. N-BF mosquitoes, we observed a different pattern: more upregulated genes (*n* = 626); YFV (*n* = 263), WNV (*n* = 29), and DNV (*n* = 334) in comparison with downregulated. The intersection between all conditions shows a higher number of upregulated genes (*n* = 131) compared to repressed genes (*n* = 54) (Figure 2B). The most altered genes were *AAEL014672* and *AAEL000870* in YFV-infected samples, *AAEL000611* and *AAEL011460* in DENV-infected samples and *AAEL011669, AAEL000611, AAEL003012* in WNV-infected samples (Figures 2A,B).

### DEGs Obtained from Infected Mosquitoes Can Discriminate Virus Infection in N-BF and BF Mosquitoes

**Figure 3:**
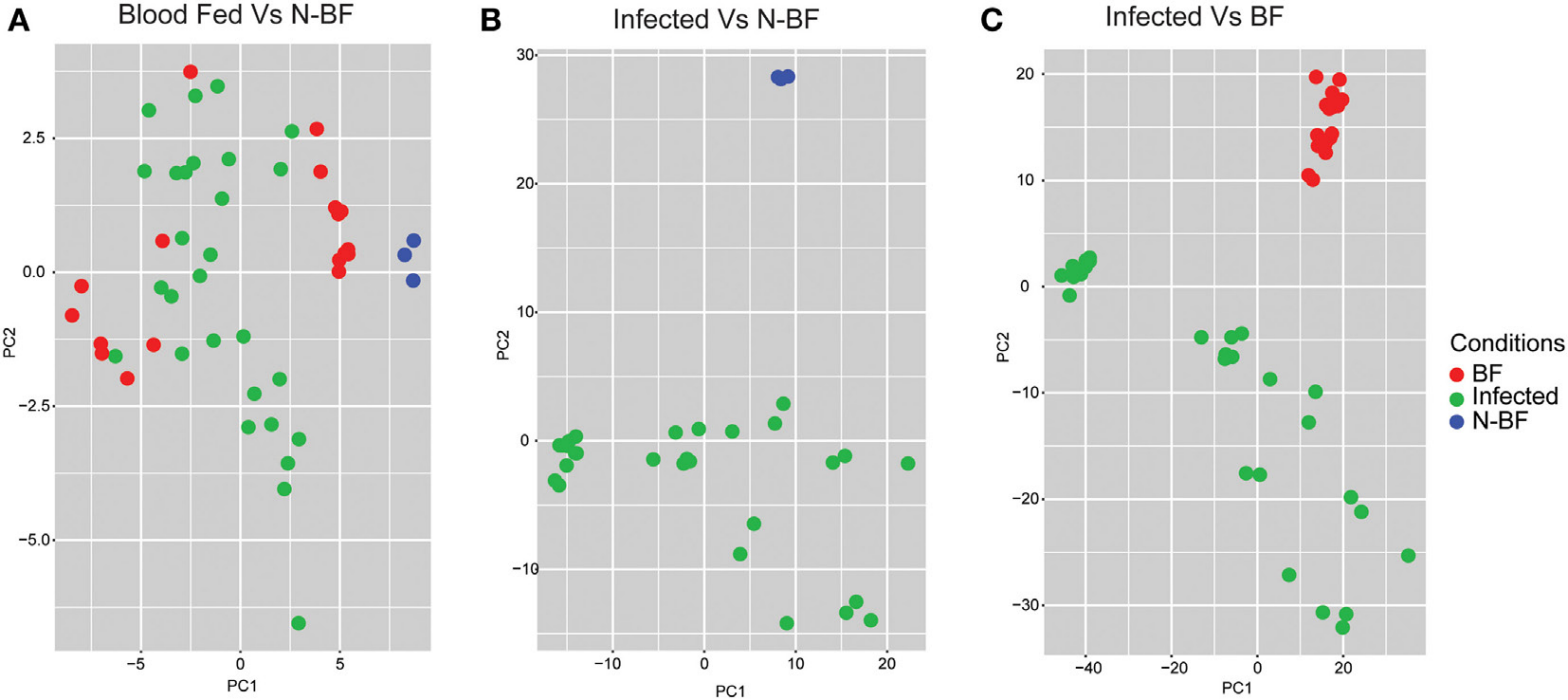
Principal component analysis of differentially expressed genes (DEGs). **(A)** DEGs from the comparison between blood-fed (BF) vs. nonblood-fed (N-BF) did not separate the BF (red), N-BF (blue), and infected (green) groups. **(B)** DEGs from the comparison of the infected (WNV, YFV, and DENV) vs. N-BF separates the N-BF (red) from the infected (blue) groups. **(C)** DEGs from the comparison of the infected (WNV, YFV, and DENV) vs. BF mosquitoes separates the BF groups (blue) and infected (red).

After we identified the global differences between the expression profiles in infected groups (DENV, YFV, and WNV) in comparison with feeding status (BF and N-BF), we performed PCA to verify whether these significant DEGs could discriminate infected, uninfected and N-BF mosquitoes (Figure 3). First, we used the 42 DEGs observed in the comparison of BF vs. N-BF (Figure 1B); however, these genes did not allow differentiation between BF, N-BF and infected mosquitoes (Figure 3A). Next, the 1,328 DEGs from the comparison of mosquitoes infected with DENV, YFV or WNV vs. N-BF allowed separation of infected and N-BF mosquitoes (Figure 3B). Finally, the 1,410 DEGs from the comparison of infected vs. BF mosquitoes could discriminate BF from infected mosquitoes with less variance, in respect to the DEGs from the infected vs. N-BF comparison (Figure 3C).

### Modules of Coexpressed Genes Related to Infection in BF and N-BF Compose Infection-Specific Expression Patterns Independent of the Dietary Background

**Figure 4:**
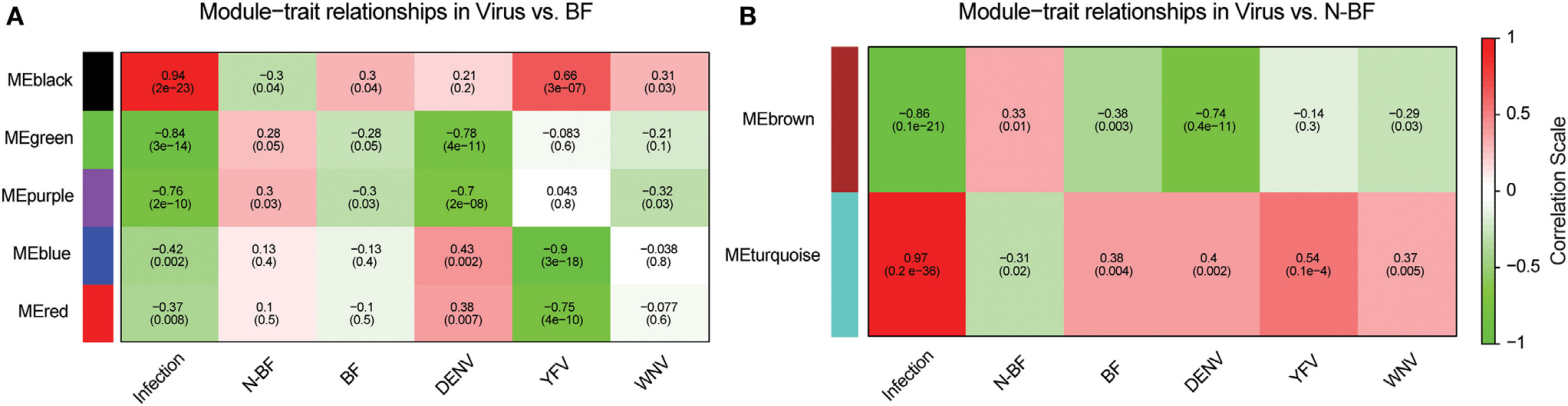
Identification of coexpression modules in infected vs. blood-fed (BF) and infected vs. nonblood-fed (N-BF) comparison. Pearson’s correlation of module eigengenes with traits (infection by each virus, BF, N-BF) are presented for each module (labeled *black, green, purple, blue, red, brown*, and *turquoise*) and corresponding *p*-value of the correlation in cells colored by the strength of the correlation. **(A)** Comparison of differentially expressed genes (DEGs) of infected vs. BF mosquitoes. **(B)** DEGs of infected vs. N-BF mosquitoes.

We constructed two weighted gene coexpression networks with WGCNA using as input the DEGs identified in the comparisons of infected (WNV, YFV, or DENV) vs. BF (1,410 genes) and infected vs. N-BF (1,328 genes) mosquitoes (Figures 2A,B). MEs, which represent the first principal component of each module, effectively summarizing its expression, were correlated with traits of interest (BF, N-BF, all infected mosquitoes, and infected by each individual virus). The first network was constructed using the DEGs from the comparison of infected vs. BF mosquitoes, resulting in five coexpression modules (color-labeled *black, green, purple, blue*, and *red*) (Figure 4A). The *black* ME showed a strong, positive correlation with viral infection (*r* = 0.94; *p*-value < 0.0001) as well as with infection by each individual virus, as expected (Figure 4A). The second coexpression network was constructed from the DEGs arising from the comparison of infected vs. N-BF mosquitoes, allowing the identification of two modules (labeled *turquoise* and *brown*). Module *turquoise* eigengene also had a strong, positive correlation with viral infection (*r* = 0.97; *p*-value < 0.0001) (Figure 4B).

**Figure 5:**
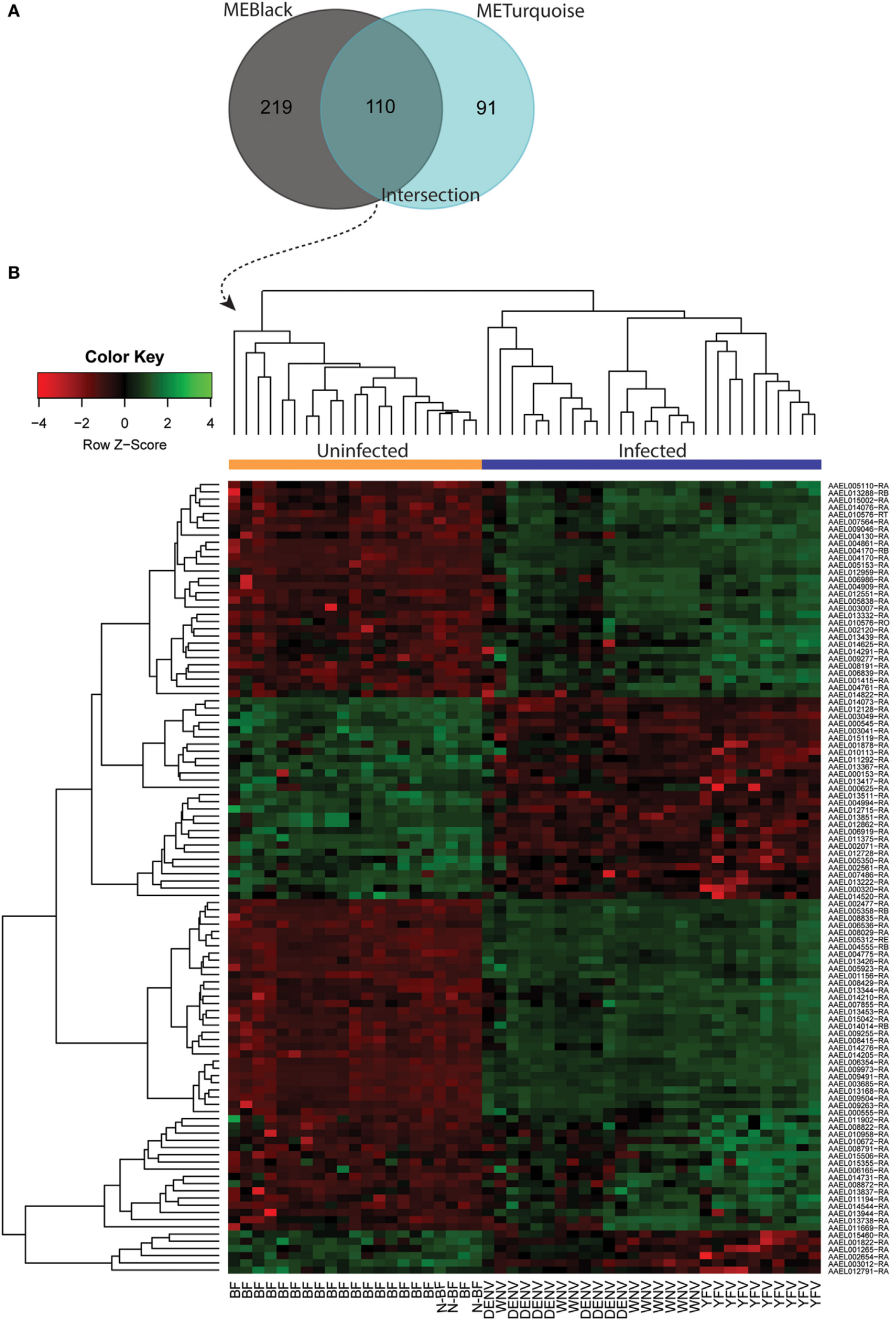
Infection-specific genes identification by removing the blood-feeding noise. **(A)** Venn diagram with genes present within the *black* and *turquoise* modules. **(B)** Hierarchical clustering and heatmap of 110 genes present in the intersection.

Both *black* and *turquoise* were the most significant modules correlated with viral infection in each comparison. Next, we analyzed the 329 genes present in *black* and 201 genes grouped into the *turquoise* modules, identifying 110 genes in common (Figure 5A) (available in Table S1 in Supplementary Material). The 110 genes in the intersection of both comparisons were regarded as forming a viral pattern of infection independent of the dietary background. To verify this, we performed hierarchical clustering analysis followed by heatmap expression visualization of these 110 genes (Figure 5). This unsupervised analysis resulted in the forming of two main groups separating the infected from the uninfected samples, suggesting an important role of these genes.

### Functional Analysis of Genes in the Coexpression Modules Reveals Distinct Processes Modulated by Infection and Feeding

**Figure 6:**
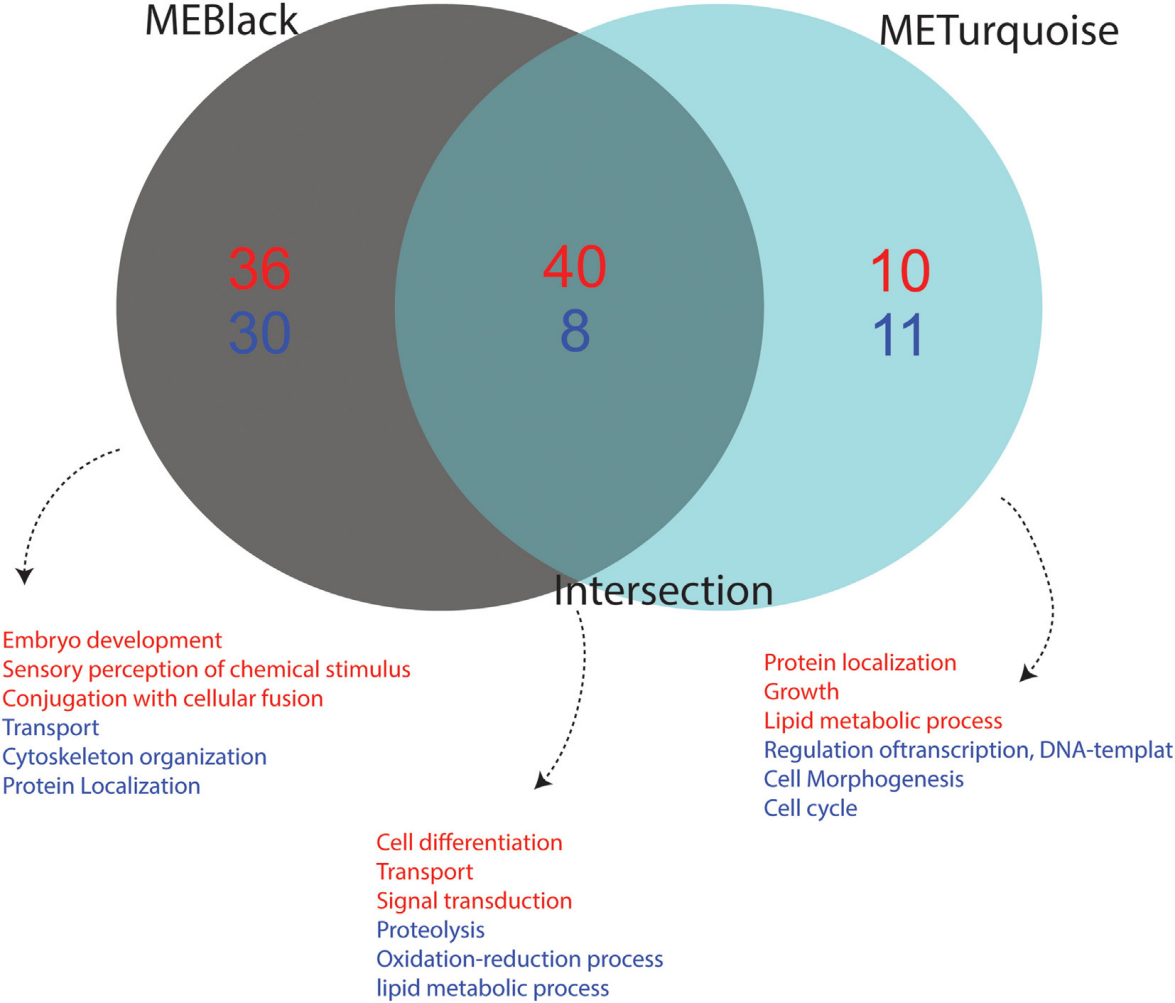
Functional annotation of up- and downregulated genes from each condition. The Gene Ontology (GO) annotation of differentially expressed genes from each module is shown. Terms in red represent upregulated functions while those in blue represent downregulated functions. Association of GO terms to genes was performed using VectorBase. The number of downregulated genes is depicted in blue and upregulated genes in red. The intersection represents the common genes between the *black* and *turquoise* modules, which corresponds to a viral-infection specific module.

We performed functional annotation of the 110 identified genes using biological processes GO terms extracted from the Vector Base repository. The main functional role of each module was summarized considering the top frequent GO term for the most variable (up- or downregulated) genes. Upregulated genes present in *black* module are mainly related to embryo development, sensory perception of chemical stimulus and conjugation with cellular fusion, while genes related to transport, cytoskeleton organization and protein localization were found downregulated. Upregulated genes in the *turquoise* module are related to “protein localization,” “growth,” and “lipid metabolic process,” while “regulation of transcription, DNA template,” “cell morphogenesis,” and “cell cycle processes” genes appeared downregulated. Finally, genes in the intersection were observed to play a role in the activation of functions related to “cell differentiation,” “transport,” and “signal transduction,” while “proteolysis,” “oxidative-reduction process,” and “lipid metabolic processes” were downregulated (Figure 6).

### Machine Learning Analysis Reduces the Infection-Specific Expression Pattern to a Small Set of Genes

**Figure 7:**
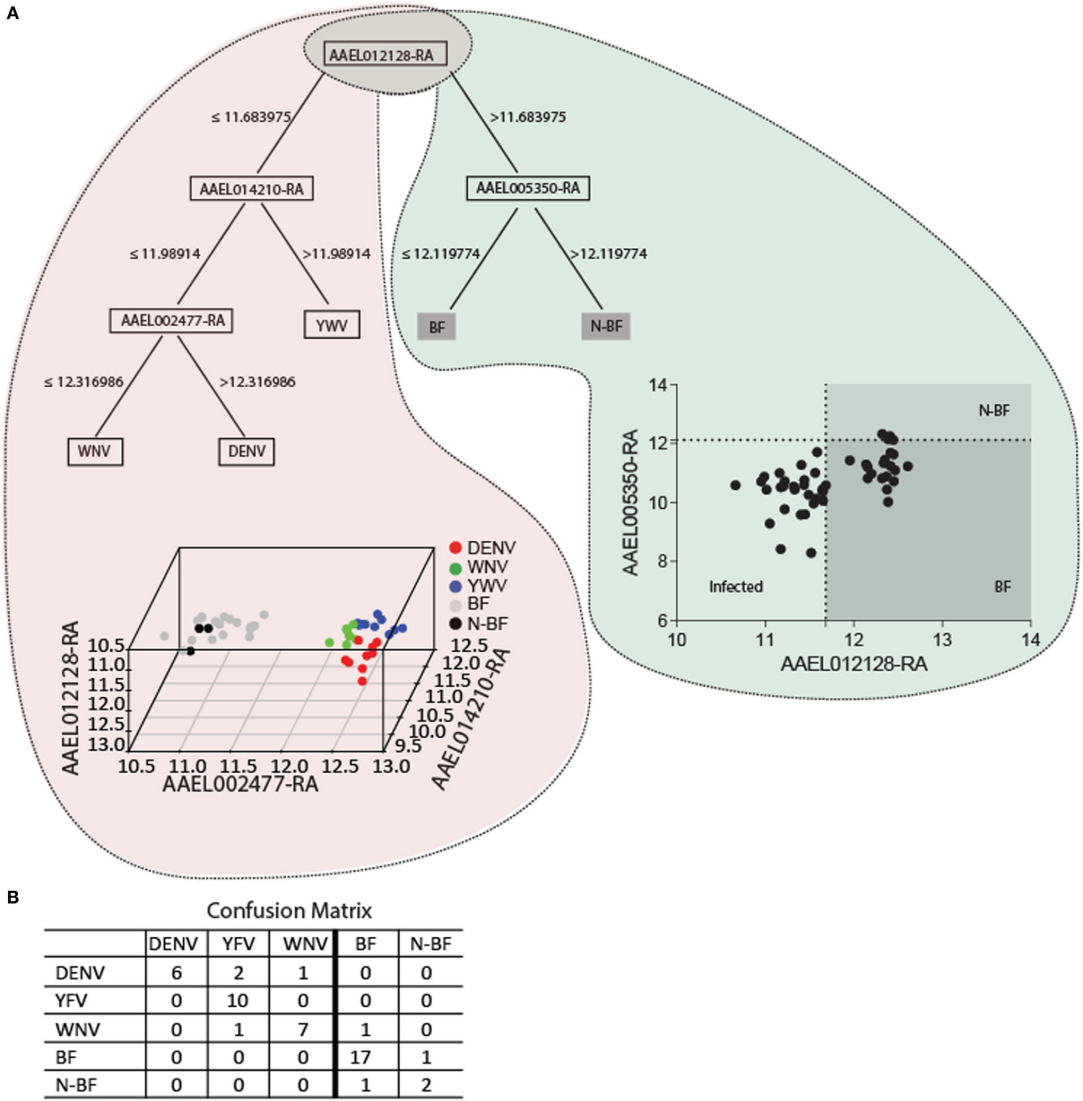
The key genes for trait classification resulting from data mining of differentially expressed genes in the modules intersection. **(A)** The J48 algorithm was used to build a decision tree using default parameters summarizes 110 genes of the intersection into four informative genes. The scatter-plot depicts by a cutoff the samples distribution discriminating each group. In the 3D plot samples are discriminated by colors according to the expression of the four genes. **(B)** The confusion matrix of the classification of the four genes estimates the classification accuracy of the decision tree. A 10-fold cross validation was performed.

We applied data mining techniques to further elucidate the importance of the genes identified in the previous analyses (Figures 5 and 6) and investigate potentially hidden connections within the gene expression datasets. A decision tree based in the J48 algorithm was constructed to identify a minimal set of genes that could explain the infection status. Four genes were able to separate infected from uninfected mosquitoes (*AAEL012128, AAEL014210, AAEL002477*, and *AAEL005350*) (Figure 7). The first gene, *AAEL012128*, classifies the mosquitoes into groups of infected (characterized by decreased expression of *AAEL012128*) and uninfected (characterized by increased expression of the gene) (Figure 7A). The remainder three genes allow further stratification of the groups according to their infection by each of the three studied viruses. The true positive rate using these genes in this classifier summed 85.71% with 7 (14.28%) incorrectly classified instances out of a total of 49, with kappa statistic (a measure of classification accuracy) of 0.8. The confusion matrix shows the number of classification errors (Figure 7B). The area under the ROC in the classification of mosquitoes infected by YFV, WNV, and DENV was, respectively, of 0.96, 0.87, and 0.83 (Figure 7B). On the other hand, area under the ROC for the classification of BF and N-BF mosquitoes was, respectively, of 0.94 and 0.82 (Figure 7B). These results pinpoint that probing the expression of a reduced number of genes in the *A. aegypti* mosquito allows the identification of its infected status.

### Independent Validation of the Role of *AAEL012128* during Infection

In order to independently validate the role of *AAEL012128*, gene identified as summarizing the infection status in our combined study, we identified two datasets related to DENV infection and verified the expression of this gene. The first dataset, reported by Sim and Dimopoulos, (2010), compared the expression of DENV-infected (live or heat-inactivated) and naïve *A. aegypti* cells (Aag2 cell line). These results are presented in Figure S1A in Supplementary Material. In line with our findings, expression of this gene was decreased in the cells exposed to the live pathogen, while samples from heat-inactivated virus had increased expression of *AAEL012128*. The second dataset, reported by Behura et al. (2011), consists of a time-course experiment comparing four DENV infected samples at 3 h and 18 h post infection (p.i.) and a control sample of RNA isolated following an uninfected blood meal. Figure S1B in Supplementary Material presents the expression of *AAEL012128* at both time-points, and a significant decrease of expression is observed at 18 h p.i. compared to the control (Mann–Whitney *U*-test; *p*-value < 0.05), as well as between the 3 and 18 h p.i. samples (Mann–Whitney *U-*test; *p*-value < 0.05), but not between 3 h p.i. and the control samples. These results show that virus presence downregulate the *AAEL012128* gene. In the other hand, to exclude the alimentation background we assessed two other datasets reported by Bonizzoni et al. (2011) and Bonizzoni et al. (2012). The first dataset used RNA-seq analyses of BF and sugar-fed mosquitoes (LVP strain) to investigate the differential gene expression in *A. aegypti* females. The second dataset used three *A. aegypti* strains, Chetumal (CTM), Rexville D-Puerto Rico, and LVP and also compared gene expression in these three strains between sugar-fed and BF alimentation regimens. The gene expression behavior of *AAEL012128* was assessed in both studies, and in line with our results this gene did not appear differentially expressed in most samples, except in the CTM strains from the Bonizzoni et al. (2012) dataset where a slight (log2FC = −0.7), but statistically significant expression decrease in BF mosquitoes was observed.

## Discussion

The establishment that the infection in a vector is influenced by multiple factors, including alimentary behavior, seasonal effects and pathogen cooccurrence is recent (Ricklefs et al., 2016). Transcriptomic analyses provide the information to better address the intermediate steps between genes and their biological roles (Wang et al., 2009). Given the myriad of diseases that can be transmitted by these vectors, identification of viral-infection markers is crucial to propose new surveillance and control strategies. Well-known pathways related to viral infection have been identified in many vectors including *A. aegypti* and *Culex quinquefasciatus* and involves activation of Toll, Imd, JAK-STAT, and RNAi pathways, which serve as defense system for controlling the infection (Gruber et al., 2008; Souza-Neto et al., 2009; Kerpedjiev et al., 2015).

However, this identification is not completely unbiased, since the infection process is closely associated with feeding behavior and consequent simultaneous modulation of genes related to both processes. In this context, we performed a combined analysis of expression data from studies that compared infected (by DENV, WNV, or YFV) and mock-infected mosquitoes (Colpitts et al., 2011) and BF against N-BF mosquitoes (Dissanayake et al., 2010). The present analysis comprises a finer-level understanding of transcriptional mechanisms associated with infection independent of the transcriptional effect due to blood feeding.

First, we investigated the similarity between the uninfected mosquitoes from the Colpitts et al. study (GSE28208), which had access to raisins as dietary source, and the BF mosquitoes from the Dissanayake et al. report (GSE22339), with both diets driving a similar expression profile (Figure 1A). However, when different diet schemes are used, we showed the existence of a high number of DEGs in the comparisons of infected vs. BF or N-BF mosquitoes (Figure 2) suggestive of influences due to an alimentation noise. PCA was applied to the DEGs arising from these comparisons, allowing the separation of infected, BF and N-BF samples, albeit with smaller variance in the comparison with the latter (Figure 3).

Next, we constructed gene expression correlation networks using DEGs from these comparisons as input. These networks have widespread use in the detection of genes that take part in common biological processes or that are regulated by an overlapping set of transcriptional factors (Kogelman et al., 2014). By constructing two separate coexpression networks (one with DEGs from infected vs. BF and another with DEGs from the infected vs. N-BF comparison) and calculating the gene content intersection in modules that related to infection (*black* and *turquoise* modules) we were able to single out 110 genes which behave infection-specific without noise interference (Figure 5A). The hierarchical clustering of these genes allowed unambiguous classification of infected and uninfected samples (Figure 5B). Interestingly, we identified in this set infection-specific expression patterns (either activation or repression) of genes playing important roles in immunity, stress and chemosensory reception. For instance, two members of the cytochrome P450 family which have been previously related to infection (Bartholomay et al., 2010; Skalsky and Cullen, 2010; Colpitts et al., 2011; Pan et al., 2012) were found among these 110 genes that discriminate infected from uninfected mosquitoes, although they were found less expressed in the infected group (*AAEL000320* log_2_FC = −1.33 and *AAEL002071* log_2_FC = −1.50, respectively). Also, genes related to immunity were identified such as *AAEL014544*, which codes for a prophenoloxidase, an insect type-3 copper enzyme involved in melanization against invading pathogens and blockade of infection (Chen et al., 2012). This gene appeared more activated in the virus-infected samples (log_2_FC = 1.72).

Also immune related was a fibrinogen-like sequence (*AAEL013417*) that was less expressed in infected samples (log_2_FC = −1.43) but which may provide a level of pathogen recognition given the lack of antibody-mediated immunity in these organisms (Dong and Dimopoulos, 2009), as well as the AMP cecropin (coded in *AAEL000625*). These peptides have been proposed as exerting both antibacterial and antiviral activities (Luplertlop et al., 2011), although expression of this gene was lower in the infected group (log_2_FC = −1.26). On the other hand, an odorant receptor coded in the gene *AAEL015506* presented the second largest expression variation relative to the uninfected group (log_2_FC = 1.95), although this change was more pronounced in the YFV-infected group. The finding that viral infection also modulates behavioral changes in the mosquito thus affecting vectorial capacity was previously reported in the context of DENV infection (Sim et al., 2012).

As a secondary result of our coexpression-based approach, we provide evidence that genes with yet unknown roles (hypothetical proteins) may actually play important parts on the infection process given their infection-specific activation patterns. One such sequence is coded in the *AAEL011669* gene, which does not have an associated function but analysis of its predicted peptide sequence reveals the presence of kinase domains (Pfam accession no. PF00069) that may be associated with the regulation of important cellular programs. Expression of this gene appeared most increased in the infected group (log_2_FC = 2.59). Another case is *AAEL013738*, also annotated as hypothetical protein. Expression of this gene was also higher in the infected group (log_2_FC = 1.82). Thus, the biological roles of these yet uncharacterized genes during infection in the *Aedes* mosquito requires further studies.

The association of biological process GO terms to the *black* coexpression module resulting from the comparison of infected versus uninfected, BF mosquitoes allowed the implication of pathways that may relate to infection and development. For instance, “embryo development” and “chemosensory perception” were both activated in the analyzed dataset. Indeed, oviposition in clean water sources starts immediately after female-mosquito feeding, and is preceded by egg maturation and site seeking (Davis et al., 2016). On the other hand, we identified decreased expression of genes that grouped to “transport,” “cytoskeleton organization” and “protein localization” terms, suggestive of host cellular components reorganization during feeding. The *turquoise* module resulting from the comparison of infected vs. N-BF grouped activated functional terms related to “protein localization,” “growth,” and “lipid metabolic processes.” These processes were not unexpected, considering the possibility of use of alternative energy sources such as fatty acids *via* β-oxidation (Arrese and Soulages, 2010).

Decreased expression of genes related to “cell cycle,” “cell morphogenesis,” and “transcriptional regulation” was observed, indicating that metabolic arrest may occur in these conditions. Finally, the intersection of both gene sets in the *black* and *turquoise* coexpression modules form the *core* infection-specific genes. Activated functional terms of these genes included “cell differentiation,” “transport,” and “signal transduction.” This reinforces what is known about the infection process that involves viral uptake and the use of host protein machinery for self-replication and viral particle assembly (Mosso et al., 2008).

In order to further reduce the set of genes that explain virus infection, we applied a machine learning technique based in decision trees using as input the 110 virus-specific expressed genes. The proposed model includes four genes (Figure 7) that classify infected from healthy mosquitoes, with *AAEL012128* being the most informative. In *A. aegypti* this gene is predicted to code for a 12-pass transmembrane protein with cationic amino acid transporter function. Expression of this gene showed a one-fold decrease in infected mosquitoes (log_2_FC = −1.03). The finding that amino acid transporters may be targeted by viral particles, as a receptor, has been reported in other insects including the silkworm *Bombyx mori*. In this organism the deletion of a gene coding for an analogous amino acid transporter generates resistance against densovirus type 2 infection, a parvo-like virus (Ito et al., 2008). Similarly, mammalian amino acid transporters spanning 12–14 transmembranes were previously reported as retroviruses receptor (Wang et al., 1991). Although a number of *A. aegypti* amino acid transporters have been experimentally characterized to date (Umesh et al., 2003; Evans et al., 2009; Hansen et al., 2011; Boudko et al., 2015) their possible functioning as viral receptors was not evaluated, and also lacking is the study of *AAEL012128* in this context.

Given the gathered evidence, we put forward the hypothesis that this gene may play an important role in the context of virus infection in the *A. aegypti* mosquito, possibly acting as a receptor. We evaluated the expression of this gene in independent datasets (not used during our Discovery analyses) related to DENV infection, and the expression trends of *AAEL012128* are in line with our findings, being decreased in infected samples (Figure S1 in Supplementary Material) or remaining unaltered when no pathogen exposure is performed. Interestingly, in one of the evaluated datasets, a time-course experiment comparing infection at 3 h and 18 h, decreased expression of *AAEL012128* can only be perceived at 18 h. Considering that one round of DENV replication occurs at approximately 30 h (Helt and Harris, 2005), this indicates that the change in expression of this cationic transporter occurs still during the initial establishment of the infection. Additionally, this gene was not differentially expressed in two independent datasets related to feeding schedule. This confirms the role of *AAEL012128* gene as an infection mark independent of blood feeding, at least in the probed mosquito strains.

The other three genes identified through data mining, *AAEL014210, AAEL002477*, and *AAEL005350* correspond to uncharacterized proteins. The first two may play regulatory roles due to the prediction of a zinc finger, DNA-binding domain (InterPro accession no. IPR013087) in *AAEL014210* and of a basic-leucine zipper domain (InterPro accession no. IPR004827) in *AAEL002477*, while *AAEL005350* harbors retinaldehyde-binding and alpha-tocopherol transport domains (InterPro accession nos. IPR001071, IPR001251).

Our study has some limitations: although the total number of samples analyzed was high (*n* = 58), regarding the infection condition we only included an equal number of mosquitoes samples infected with DENV, WNV, and YFV from the Colpitts et al. (2011) dataset. This strictly limits the generalization of our results to genes related to infection by these viruses. While there exists others expression sets related to *A. aegypti* infection, it is not surprising that most of them focus on DENV infection, such as the works of Behura et al. (2011) and Sim and Dimopoulos (2010). This occurs given the DENV relevance in most tropical and subtropical areas worldwide. These samples were not included in the first analysis in order to avoid overrepresentation of a DENV-specific transcriptional response and were used in external validation. This overrepresentation could lead to biases in our gene expression correlation-based approach.

Other publicly available expression datasets related to the *A. aegypti* mosquito address more specific questions such as insecticide resistance (Kasai et al., 2014), sex differences (GEO accession no. GSE7813), developmental aspects (GEO accession nos. GSE23039, GSE7811, GSE71221, and GSE90515), and circadian mechanisms (Ptitsyn et al., 2011; Leming et al., 2014; Bottino-Rojas et al., 2015; Jupatanakul et al., 2017). For this reason these studies were not considered in our analyses. Furthermore, our dataset contains only the LVP and Rockefeller mosquito strains in the discovery dataset. During the validation step, we were able to confirm our findings in a DENV infection study that used Moyo mosquitoes as well as in LVP and D-Puerto Rico mosquitoes strains from an alimentation dataset, but not in CTM samples from this same dataset. In an Aag2 cell line infected with DENV the expression response of *AAEL012128* was also in line with our findings. Thus, there exist strain-specific differences forming part of the *A. aegypti* response to infection and to feeding in blood that limits extrapolation of gene expression findings.

## Conclusion

In this study, we performed an integrated analysis of *A. aegypti* expression datasets totaling 58 samples and validate in four different datasets. We aimed the identification of virus infection-specific gene sets independent of feeding behavior. Using a correlation-based analysis, we determined a set of 110 genes that are specific of the vector response to the viral infection. Further reduction of this dataset allowed the identification of four genes with high information gain on discriminating infected mosquitoes, and these were validated using independent datasets. Our derived, integrated dataset of *A. aegypti* transcripts could orientate experimental confirmation of the role of the identified genes during viral infection. Increased knowledge on the transcriptomic aspects specific to the infected mosquito could be a means to the design of novel vector control strategies and better understanding of vector biology during infection.

## Availability of Data and Materials

All data analyzed during this study are already publicly available or included in this article as Additional files. The original data are available in the Gene Expression Omnibus^3^ under accession nos. GSE28208 and GSE22339 and associated manuscripts. Data used during validation are also deposited in GEO under accession nos. GSE16563, GSE33274, or within the supporting files of the respective manuscripts (http://www.g3journal.org/content/2/1/103.supplemental; and https://www.ncbi.nlm.nih.gov/pmc/articles/PMC3042412/bin/1471-2164-12-82-S2.XLSX). The derived results obtained during our analyses (intersection of coexpression modules) supporting the conclusions of this manuscript are included within the text and in the associated supplementary material.

## Author Contributions

AQ, PR, and KF conceived of the study. KF, JK, PR, AP, and AQ performed data analysis. AQ, KF, PR, AB, CO, and MG drafted the manuscript with input from the other authors. All authors read and approved the final manuscript.

## Conflict of Interest Statement

The authors declare that the research was conducted in the absence of any commercial or financial relationships that could be construed as a potential conflict of interest.

## Footnotes

^1^http://www.ncbi.nlm.nih.gov/geo/.

^2^https://www.vectorbase.org.

^3^http://www.ncbi.nlm.nih.gov/geo.
