# Supplementary material for: Meta-Analysis of Aedes aegypti Expression Datasets: Comparing Virus Infection and Blood-Fed Transcriptomes to Identify Markers of Virus Presence
Source: Front Bioeng Biotechnol. 2018 Jan 11;5:84. doi: 10.3389/fbioe.2017.00084 (PMC5768613; doi:10.3389/fbioe.2017.00084)
Supplement: Supplementary file 1 [file Image_1.PDF]

Supplementary Material

Meta-analysis of Aedes aegypti expression datasets: comparing virus infection and blood-fed transcriptomes to identify markers of virus presence.

Kiyoshi Ferreira Fukutani [a,b,§,] José Irahe Kasprzykowski [a,c,§,] Alexandre Rossi Paschoal [d,e,] Matheus de Souza Gomes [f,] Aldina Barral [a,g,] Camila I. de Oliveira [a,g,] Pablo Ivan Pereira Ramos [a,#,] Artur Trancoso Lopo de Queiroz [a,c,h,#]

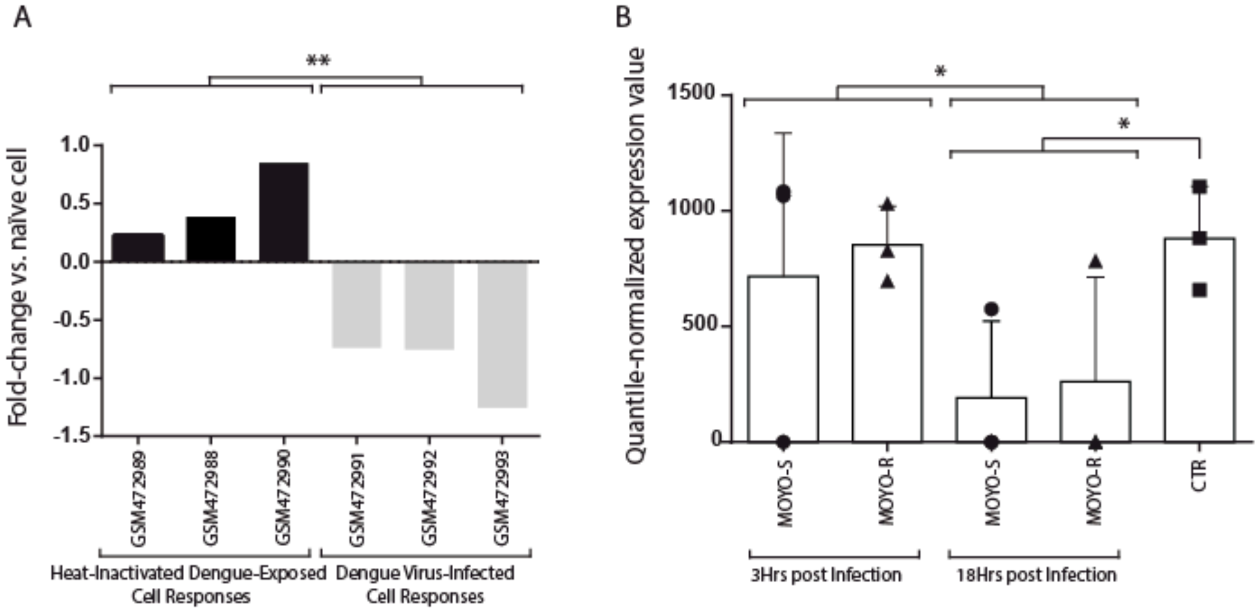

Supplementary Figure 1. Expression of AAEL012128 in two independent datasets. Panel A, evaluation of the expression of AEG_V1.13480 (probe name for AAEL012128 in this platform) in the *Ae. aegypti* Aag2 cell line of heat-inactivated DENV exposed cells (GSM472988, GSM472989 and GSM472990) and DENV-infected cells (GSM472991, GSM472992 and GSM472993). Vertical bars represent fold-changes compared to naïve cells. Panel B, expression of the gene in a time-course experiment comparing four DENV infected samples at 3h and 18h p.i. (MOYO-S, an *Ae. aegypti* strain susceptible to infection; MOYO-R, a strain more refractory to infection) and a control sample that consisted of RNA isolated following an uninfected blood meal. Vertical bars represent quantile-, RMA-normalized expression values. *, Mann-Whitney U test, p-value <0.05; **, Mann-Whitney U test, p-value <0.01.
